# Hyperthermia combined with immune checkpoint inhibitor therapy: Synergistic sensitization and clinical outcomes

**DOI:** 10.1002/cam4.5085

**Published:** 2022-07-31

**Authors:** Pengyuan Liu, Mengna Ye, Yajun Wu, Lichao Wu, Kaiping Lan, Zhibing Wu

**Affiliations:** ^1^ Oncology & Radiotherapy Department Zhejiang Hospital Hangzhou China; ^2^ Second Clinical Medical College Zhejiang Chinese Medical University Hangzhou China; ^3^ Department of TCM Pharmacy Zhejiang Hospital Hangzhou China; ^4^ College of Basic Medical Sciences Zhejiang Chinese Medical University Hangzhou China; ^5^ Oncology Department of Combination of Traditional Chinese and Western Medicine Tonglu Hospital of Traditional Chinese Medicine Hangzhou China

**Keywords:** hyperthermia, immune checkpoint inhibitor, synergistic sensitization, tumor immunosuppression

## Abstract

**Background:**

Within the field of oncotherapy, research interest regarding immunotherapy has risen to the point that it is now seen as a key application. However, inherent disadvantages of immune checkpoint inhibitors (ICIs), such as their low response rates and immune‐related adverse events (irAEs), currently restrict their clinical application. Were these disadvantages to be overcome, more patients could derive prolonged benefits from ICIs. At present, many basic experiments and clinical studies using hyperthermia combined with ICI treatment (HIT) have been performed and shown the potential to address the above challenges. Therefore, this review extensively summarizes the knowledge and progress of HIT for analysis and discusses the effect and feasibility.

**Methods:**

In this review, we explored the PubMed and clinicaltrials.gov databases, with regard to the searching terms “immune checkpoint inhibitor, immunotherapy, hyperthermia, ablation, photothermal therapy”.

**Results:**

By reviewing the literature, we analyzed how hyperthermia influences tumor immunology and improves the efficacy of ICI. Hyperthermia can trigger a series of multifactorial molecular cascade reactions between tumors and immunization and can significantly induce cytological modifications within the tumor microenvironment (TME). The pharmacological potency of ICIs can be enhanced greatly through the immunomodulatory amelioration of immunosuppression, and the activation of immunostimulation. Emerging clinical trials outcome regarding HIT have verified and enriched the theoretical foundation of synergistic sensitization.

**Conclusion:**

HIT research is now starting to transition from preclinical studies to clinical investigations. Several HIT sensitization mechanisms have been reflected and demonstrated as significant survival benefits for patients through pioneering clinical trials. Further studies into the theoretical basis and practical standards of HIT, combined with larger‐scale clinical studies involving more cancer types, will be necessary for the future

## Graphical abstract

Our review analyses and discusses the recent basic research and clinical application of hyperthermia combined with immune checkpoint inhibitors for malignant tumors treatment. And the development tendency of this therapy is set forth constructively, which aims to provide a helpful guide to follow‐up practice.

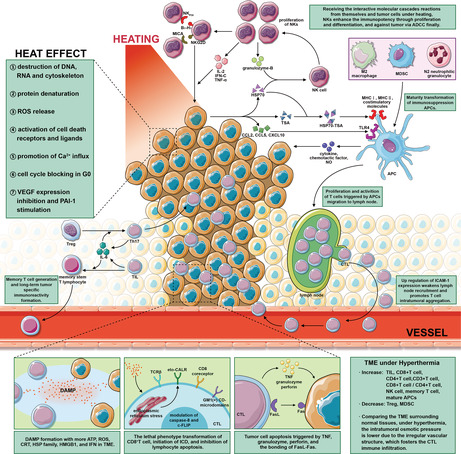

## 1 INTRODUCTION

Under the influence of social factors such as poor lifestyle, environmental modification, and aging populations, cancer remains a recalcitrant disease that seriously impacts global public health.^1^ According to the World Cancer Report, in 2020 there were 19.29 million new cancer cases and 9.96 million cancer‐related deaths.^2^ Long‐term endeavors have brought limited breakthroughs regarding conventional treatments for cancer recurrences and metastasis.^3^ Remedial innovations remain the main source of solutions to overcome traditional therapeutic barriers.^4^

Immunotherapy has developed over the past decades through the mass exploration of tumor immunology; it has become a promising strategy for combating cancer.^5^, ^6^ It can now be asserted that immunotherapy has grown from a potential topic of interest into a viable, important application.^7^ This was demonstrated in 2018, when James P. Allison and Tasuku Honjo were awarded the Nobel Prize in Physiology or Medicine, providing strong evidence that immune checkpoint inhibitor (ICI) treatment has gained mainstream recognition.^8^ Indeed, the application of anti‐PD‐L1/PD‐1 and anti‐CTLA‐4 for metastatic melanoma and lung cancer are now included in the National Comprehensive Cancer Network (NCCN) guidelines.^9^ In the 10 years since the first ICI, Ipilimumab was approved by the FDA for unresectable or metastatic melanoma, numerous clinical studies have demonstrated that ICIs provide substantial survival benefits to patients in some cancer subtypes such as Hodgkin's lymphoma, Merkel cell carcinoma, desmoplastic melanoma, or at the tumors with high PD‐L1 expression, MSIH/pMMR, and high TMB molecular phenotypes.^10^, ^11^ ICI can exert an excellent 50%–90% efficiency in these cancer types,^12^ but these conditions are relatively rare. In contrast, the response rate to ICI is only about 15%–25% for cancers covering most population, including most lung cancers, MSIL/MSS/dMMR gastrointestinal cancers, breast cancers, prostate cancers, hepatocellular carcinomas, head and neck cancers, urothelial carcinomas, renal cell carcinomas, etc.^13^, ^14^, ^15^, ^16^ In addition, it should also be noted that the development of acquired resistance to ICI remains unavoidable, which is associated with tumor‐mediated immunosuppression, defective antigen delivery, neoantigen depletion, and loss of interferon‐γ (IFN‐γ) signaling, and upregulation of IC bypass expression.^17^ Therefore, ICI still has significant limitations at present.

Solving the predicaments encountered during traditional or single therapies could contribute to the development of a comprehensive oncotherapy model.^18^ Currently, improved combined treatments are being collocated by clinicians through the selection of combinations of imperfect treatments.^19^ For locally advanced gastrointestinal malignancies that are difficult to surgery directly, so neoadjuvant chemotherapy strategies have been proposed in recent decades, which greatly improve the feasibility of surgery and R0 resection rate, thus significantly prolonging survival.^20^ For pancreatic cancer with highly prone to recurrence after surgery, the pioneering ESPAC‐1 study explored the effects of adjuvant chemotherapy.^21^ The results showed that 5‐fluorouracil combined with calcium leucovorin improved the 5‐year OS rate to 21%, compared to only 8% with surgery alone. A Meta‐analysis involving 1086 cases of NSCLC with brain metastases showed that radiotherapy combined with TKI significantly improved intracranial PFS and OS compared to TKI alone.^22^ These studies combine the advantages and settle the disadvantages of different remedies, which encourage cancer comprehensive treatment. Strategies that aid the selection of congenial remedies to cover shortcomings and add advantages for ICIs are urgently required; significant efforts are now being made in this regard.^23^, ^24^

Physiologic fever is normally a crucial function that improves body immunity.^25^ Hyperthermia, which entails being subjected to external heat sources, is a novel oncotherapy that can release steerable thermal energy to modulate intratumoral immunology, and thus potentially cure cancer.^26^ Through developments in medical physics, the clinical operability, precision, and universality of hyperthermia are all currently increasing.^27^, ^28^ With the widespread availability of hyperthermia, the amelioration in cancer immunosuppression has been observed in many basic studies,^29^, ^30^ leading to the exploration of the potential for combining hyperthermia with ICI treatment (HIT). Emerging results in this regard have not only verified and enriched the theoretical foundation of HIT sensitization but are also now gradually transitioning the development stage of HIT from preclinical explorations to clinical investigations.^31^, ^32^, ^33^

Though some articles have pertinently analyzed the mechanism of HIT by focusing on different cells, immunological mediators, and systemic immune status in mice,^9^, ^34^, ^35^ to date there has been no comprehensive review into the connectivity between basic and clinical research, or between animal and human studies. Here, therefore, studies into HIT mechanisms featuring animal models are reviewed, and the molecular and cellular changes are traced through an analysis of their HIT curative efficacy. In addition, new clinical works with different classifications are addressed, and likely future trends in the development of HIT are discussed. This review aims to provide a helpful guide to follow‐up practices regarding HIT.

## 2 MECHANISM AND RESISTANCE OF ICIs

**FIGURE 1:**
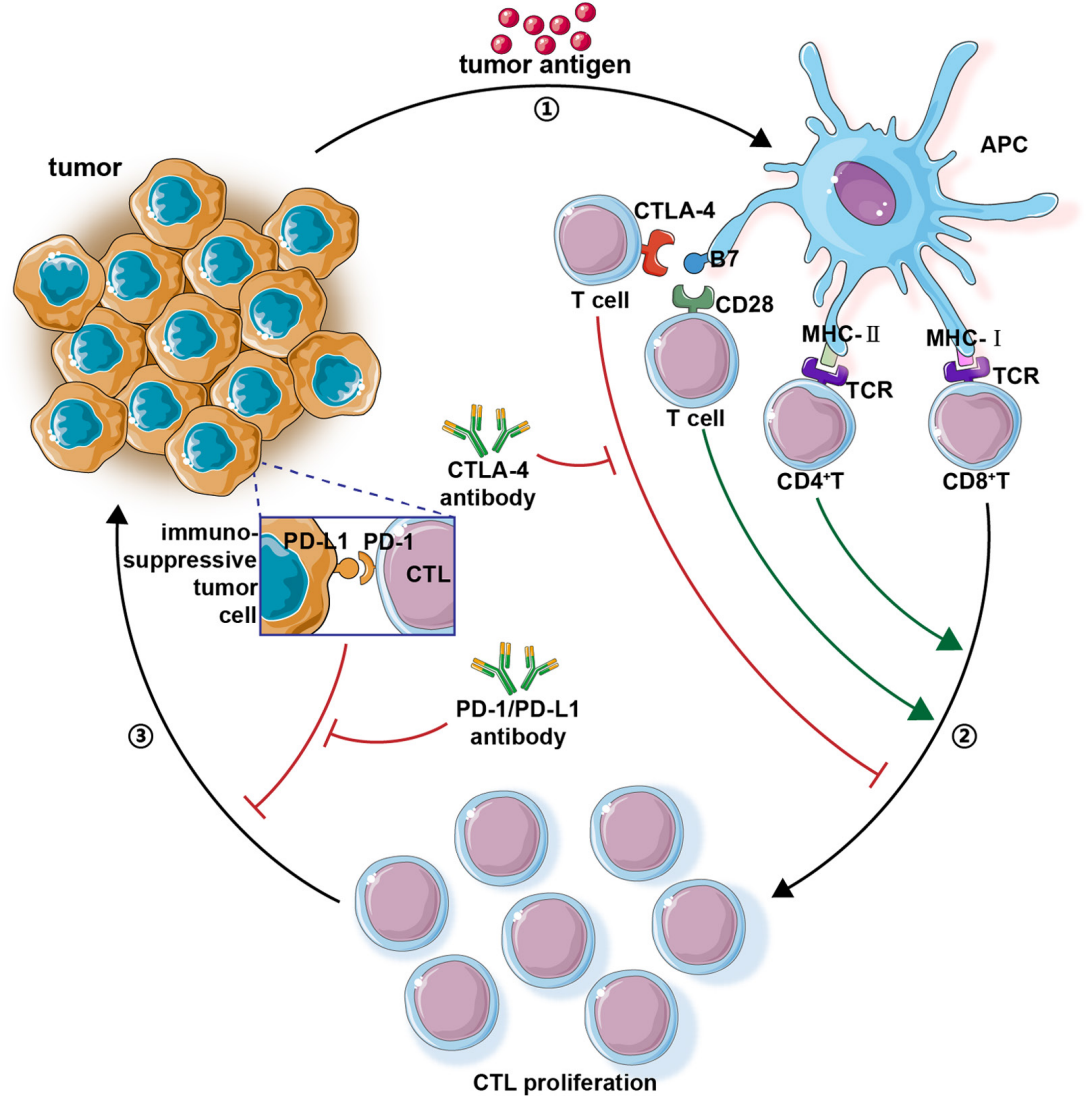
Schematic of the mechanisms of PD‐L1/PD‐1 and CTLA‐4 inhibitors. ①: The tumor antigens are taken up and processed by APC and presented to CD4+T cells and CD8+T cells. ②: The activation and proliferation of CD8+T cells can be triggered by APC through the combination of MHC‐I and TCR; CD4+T cells stimulated by APC promote this process. Meanwhile, CD28 and CTLA‐4 on T cells competitively bind to the B7 ligand expressed on APC; the former can facilitate the activation and proliferation of CD8+T, while for the latter the opposite is true. This immunosuppression can be antagonized by CTLA‐4 inhibitors. ③: Immune killing by increased and activated CD8+T cells elicits more antigens, which achieves positive feedback. The overexpression of PD‐L1 in immunosuppressive tumor cells can impede the immunopotency of CTL; PD‐1/PD‐L1 inhibitors could remit this.

Negative regulation, led by immune checkpoints (ICs), maintains a balance between sensitive exclusion and damage to innocent cells. This regulation can preserve the appropriate immune state under normal circumstances. However, mutated tumor cells can increase the expression of some proteins, such as the up‐regulation of PD‐L1 expression, which inhibits the killing effect of cytotoxic T‐lymphocyte by promoting its binding with PD‐1, thus resulting in “immune escape.”^36^ Based on the above mechanisms, ICIs aim to relieve part of the immunosuppressive signaling pathway and restore the vigor of immune cells.^37^ The mechanisms of two ICIs (PD‐L1/PD‐1 and CTLA‐4) are summarized briefly in Figure 1.

Although ICIs have developed into a promising research direction following rapid development in recent years, overall clinical response rates range from 15% to 60%, suggesting there is a loss in efficacy in practical scenarios compared to theoretical predictions.^38^ ICIs operate within the tumor microenvironment (TME) under the influence of intratumoral heterogeneity and cellular mutability. This could result in there being an insufficient number of local tumor‐infiltrating T cells (TILs), thus decreasing the killing effect of TILs and leading to difficulties regarding the formation of memory T cells. These pathways can inhibit ICIs, leading to a “drug‐resistant TME.”^39^, ^40^ In 2015, Allison first proposed the concept of immunogenic and non‐immunogenic tumors (“hot tumors” and “cold tumors”). The former refers to the presence of high numbers of TILs and interferon‐gamma (IFN‐γ)^+^T in the TME, combined with the high expression of PD‐L1 in tumor cells; the latter refers to the opposite.^41^, ^42^ In addition to the gene mutation of tumor cells, whose TMEs can worsen the phenotype, independent of other directions, mutations can affect ICIs, host immunity, and intestinal microecology. They can also reduce the clinical effectiveness of ICIs.

## 3 HYPERTHERMIA

Hyperthermia is a kind of physical therapy that uses the biological thermal effect and physical factors of non‐ionizing radiation to heat tissue, thus killing tumor tissue or promoting tumor cell apoptosis.^43^ It can be divided into whole‐body hyperthermia and local hyperthermia according to its scope, and into radiofrequency, ultrasound, microwave, infrared, intracavitary perfusion, and nanoparticle hyperthermia according to the heating factor being used.^44^ Thus, hyperthermia is not only flexible regarding its heating form but also regarding its different curative purposes, thus deriving dual pattern hyperthermia.^45^

Ultra‐hyperthermia (UH) refers to thermal ablation, in which the temperature often rises above 50°C; it is used to kill carcinoma directly. UH includes microwave/radiofrequency ablation, high‐intensity focused ultrasound (HIFU), and nanomaterial‐based hyperthermia.^46^ Meanwhile, mild hyperthermia (MH) is conducted at temperatures below 45°C; it is mainly used to assist oncotherapy. MH utilizes the pathophysiology of inordinate vessels and unsound thermoreceptors, which can yield a difference of 3°C between neoplasm and normal tissue.^47^ Regarding the anti‐tumor performance of hyperthermia, UH should mainly be applied to the irreversible denaturation of proteins, whereas induced apoptosis through MH might be more suited to impairing malignancies. The different curative purposes of UH and MH determine the different indications. In short, UH is more suitable for solid tumors with oligometastasis or local progression that are difficult to remove surgically, and some patients can achieve the same results as surgery by UH. For example, thermal ablation for patients with a single lesion ≤5 cm in diameter or for patients with 2–3 lesions and the largest lesion ≤3 cm in diameter in hepatocellular carcinoma can be curative, and the adverse events are significantly lower than those of surgery.^48^ While MH prefers patients with advanced metastatic cancer, such as intracavity hyperthermia chemotherapy mainly for multiple metastases in the peritoneum, pleura, bladder, and other tissues, especially those who have developed malignant effusions. The membrane permeability of cancer cells can be increased by MH which facilitates the entry of chemotherapeutic drugs to exert cytotoxic effects.^49^ Moreover, whole‐body hyperthermia induces thermogenic factors to systemic thermal stress, which greatly mobilizes the host immunostimulation. This could be a potent adjuvant regimen for comprehensive medication to be suitable for extensive metastasis or patients receiving palliative treatment. In general, the indications of hyperthermia are quite flexible due to the variety of modalities and types of heating, and physicians can choose hyperthermia mode according to the equipment available in their institutions.

Among the multitudinous combination treatments program that includes hyperthermia, it is important to note that HIT is fundamentally different from ICI combined with conventional therapy. Though chemotherapy^8^ and radiotherapy^50^ are both also immunostimulatory, the cytotoxicity difference between conventional treatments and hyperthermia regarding normal immunity could lead to the latter having a relatively positive impact on tumor immunology, unlike other radical therapies. Though the limited damage that hyperthermia causes to immunization would not compensate for its initial influence on immunoreactivity, it could help to form a curative immunosensitive TME to aid ICI activation.

## 4 SYNERGISTIC SENSITIZATION OF HIT

**FIGURE 2:**
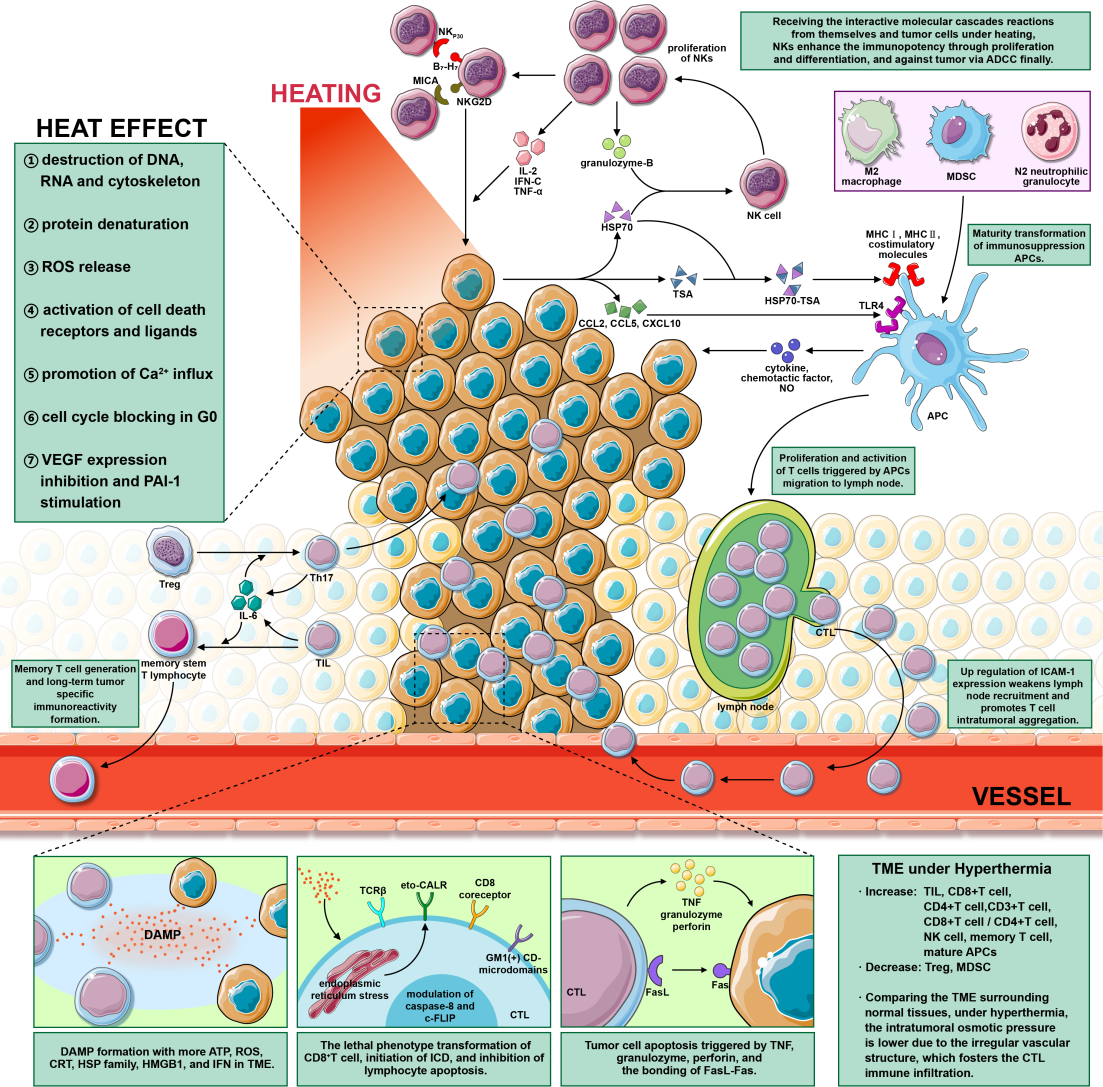
Guided by seven heat effects, a battery of lethal and sublethal biologic alterations in tumor cells interact with immunocytes to induce the generation of DAMP. The number of immune‐activated subtypes and the tumor‐killing potency of NK cells is greatly augmented in the TME under DAMP.

The pharmacologic actions of ICIs can be enhanced by applying thermal stress to the IC axis; this is called direct sensitization. Indirect sensitization, meanwhile, comprises a series of positive immunoregulation paralleling direct mechanisms. The fact that heat can have these effects independent of immunostimulation further reinforces the power of HIT against carcinoma. Several key mechanistic pathways in HIT synergistic sensitization are shown in Figure 2.

### 4.1 Direct sensitization

Oncologists are currently expanding the theoretical cognitive dimensions of ICs, which is, in turn, creating further opportunities for thermophysical biology to provide new entry points to direct sensitization.^51^ Hyperthermia can trigger a series of multifactorial molecular cascade reactions between tumors and immunization, thereby significantly inducing cytological modifications within the TME.^52^ The immunomodulatory amelioration of immunosuppression, combined with the activation of immunostimulation, can greatly enhance the pharmacological potency of ICIs.

Both tumor cells and dendritic cells (DCs) can transform intracellular and extracellular secretion under thermal stimulation. These processes promote the activation and maturity of antigen‐presenting cells (APCs), leading to their differentiation into subtypes, accompanied by the modification of T cell phenotypes.^53^, ^54^, ^55^ For example, it has been shown that the differentiation of T lymphocytes into Th2 and regulatory T cells (Tregs) in the spleen can be restrained by heating at 39–40°C; the multiplication of Th1 and Tc1 cells increases under these conditions.^56^ Within the human body, Tregs have also been shown to differentiate into Th17 under IL‐6 due to hyperthermia.^57^

The immunopotency of CD8^+^T cells can be enhanced by the membrane aggregation of GM1(+) CD‐microdomains, T‐cell receptor (TCR)β, and CD8 coreceptors, as well as by the secretion of IFN‐γ under heating.^58^ The translocation of AP‐1 and NK‐κB can also increase the expression of Fas ligand due to up‐regulation by heat shock factor‐1 (HSF‐1), which ultimately offers a potent killing efficiency.^59^

Immunogenic cell death (ICD) is an influential part of HIT synergism. Based on the generation of tumor neoantigens, calcium reticulin surface exposure (eto‐CALR) can be triggered by the production of reactive oxygen species (ROS) and endoplasmic reticulum stress through hyperthermia; this is a prerequisite of ICD.^60^ Following the secretion of IFNs and adenosine triphosphates (ATPs), the intratumoral immune microenvironment can develop to form damage‐associated molecular patterns (DAMPs). This involves the preponderance of calreticulin (CRT), heat shock protein‐70 (HSP70), heat shock protein‐90 (HSP90), and high mobility group box 1 (HMGB1).^61^

The formulation of memory stem T cells can also be increased by hyperthermia, which may be attributed to the mass release of IL‐6.^62^, ^63^ The curative effect analysis described below revealed that long‐term tumor suppression through the generation of anamnestic immunity is crucial for controlling recurrence and prolonging survival.

ICI response rates have been shown to be positively correlated with the tumor mutation burden (TMB).^64^ Hyperthermia can enhance the TMB by damaging deoxyribonucleic acid (DNA) and impelling the release of neoantigens. Thus, it could guide both specific and non‐specific immunity to a large extent.^65^ Rangamuwa et al.^66^ observed that 80% of patients treated with bronchoscopic thermal vapor ablation exhibited up‐regulated expressions of PD‐L1. This suggests and possibly validates the ability of hyperthermia to transform “cold tumors” into “hot tumors.” Meanwhile, the quantities of Treg^67^ and Th17^57^ have been shown to be noticeably decreased after hyperthermia, which can relieve immunosuppression to some extent. The multiple actions highlighted above could eventually contribute to both neoplastic vulnerability and immunizing aggressivity, thus offering valid ICI pharmacodynamics that can be inferred and observed. It is possible to benefit from these multifactorial mechanisms by enhancing ICI‐mediated immunopotency and unburdening immunosuppression. Thus, the promotion of ICI efficacy by hyperthermia may be observable.

### 4.2 Indirect sensitization

Indirect sensitization is an immunologic supplement for ICIs. Biological modifications in APCs can augment specific immune functions, such as enhancing the immunoreactivity of cytotoxic T lymphocytes (CTLs). Furthermore, phenotypic remolding can regulate tumor nonspecific immunization.^68^

The mature transformation of immunosuppressive APCs such as M2 macrophages, N2 neutrophilic granulocyte, and myeloid‐derived suppressor cells (MDSCs) can arise in hyperthermia above 43°C.^69^ Natural Killer (NK) cells are currently being studied regarding HIT nonspecific immunization. The proliferation, migration, and cytotoxicity of NK cells can be greatly enhanced by varying their molecular expression, distribution, and secretion via heating.^70^, ^71^ Furthermore, substantial endogenous antigens are released under thermal damage, which can cause natural host vaccines to recruit more antibody‐dependent cell‐mediated cytotoxicity (ADCC) due to the preponderance of NK cells.^72^

## 5 PRECLINICAL STUDIES OF HIT

### 5.1 Ultra‐hyperthermia combined with ICI treatment

In recent years, numerous animal experiments have combined ablation with ICI, their results can serve as a link between basic and clinical studies into HIT. Several of the preclinical studies are summarized in Table 1. In the mouse colon cancer CT26 and melanoma B16 cell models, Shi et al.^73^ found that the proportion of CD8^+^T/Tregs increased significantly, as did the secretion of IFN‐γ and tumor necrosis factor‐α (TNF‐α) by TILs. The lethality of ablation is much stronger than that of MH, so it can create more endogenous antibodies. Follow‐up results showed that tumor volume was significantly controlled in HIT mice, for which the highest survival rate was achieved. The host immune hyper‐responsiveness caused by UH could be the main reason for the abovementioned increases in molecules and cells. Han et al.^74^ carried out HIFU/RFA and anti‐CTLA‐4 combined with adjuvant experiments in mice to verify the control of metastases after primary lesion ablation. Combined with the findings of Shi et al.^73^ not only did the amount of Treg decrease, but the number of MDSCs in the TME also appeared to decline. Han et al.^74^ also proposed that HIFU/RFA increased the uptake of tumor‐specific antigen (TSA) by DCs, which is a common result of antigen exposure and DC aggregation induced by hyperthermia. Their data showed that the CTLs of metastatic lesions increased to 17.31%, while the Treg ratio (31.67%) was significantly lower than that in an untreated state (54.05%); the ratio of CD8^+^T/Tregs increased fivefold. The distant metastasis of the HIT group, compared with a group that underwent ablation alone, subsided significantly, and there were no obvious adverse reactions.

**TABLE 1:** HIT pre‐clinical studies

Reference	Treatment	Tumor model	Microscopic foundation	Curative advantage in vivo
[73]	Radio‐frequency ablation (RFA) Anti‐PD‐1 (clone J43)	CT26 cell B16 cell	RFA increases TIL number and CD8^+^/CD4^+^ ratio in TME RFA significantly increases the Teff/Treg ratio. RFA upregulates PD‐L1 expression in tumor cells, even for distant mock metastases without ablation. HIT enhances the expression of IFN‐γ and TNF‐α.	HIT significantly reduced the tumor size and prolonged survival in mice.
[74]	HIFU/RFA Anti‐CTLA4 (Clone 9D9) Immune adjuvant (TLR7/TLR4 agonist)	CT26 cell	Hyperthermia significantly increases the proportion of mature DC subtypes (CD11c^+^, CD80^+^, CD86^+^). HIT with adjuvant reduces the number of Treg and MDSC. HIT with adjuvant increases the proportion of CTL in TME. HIT with adjuvant induces the generation of T memory cells (CD3^+^, CD8^+^, CD44^Hi^, and CD62^Lo^).	Mice in the HIT combined with the adjuvant group achieved 100% survival at day 40 and their distant metastasis model disappeared, and mice in this group had the highest survival rate after simulated recurrence.
[75]	Magnetic hyperthermia Anti‐PD‐L1 (Bio X Cell, Catalog: BE0101)	4 T1 cell	Hyperthermia stimulates the release of tumor‐associated antigens. Hyperthermia significantly increases the proportion of mature DC subtypes (CD11c^+^, CD80^+^, CD86^+^). Hyperthermia increases the CD8^+^T number in TME. Hyperthermia increases the expression of IFN‐γ and TNF‐α.	The growth of simulated distant metastases was significantly inhibited in the HIT group. The 60‐day survival rate of mice in the HIT group was 83%, while 0% in the rest of the groups.
[76]	Photothermal therapy Anti‐PD‐L1 Photodynamic therapy Chemodynamic therapy	CT26 cell	Hyperthermia increases the amount of calreticulin in TME and consequently induces ICD. Hyperthermia increases the expression of IL‐6, IFN‐γ, and TNF‐α.	HIT prevented lung metastases and prolonged survival.
[77]	Photothermal therapy Anti‐PD‐1 peptide	CT26 cell 4 T1 cell	Hyperthermia increases the number of CD3^+^T, CD8^+^T, and CD4^+^T in TME. Hyperthermia increases the expression of IL‐2, IFN‐γ, and TNF‐α while decreasing IL‐10.	HIT prevented distant metastases and prolonged survival.
[78]	Photothermal therapy Anti‐PD‐L1 (Bio X Cell, Catalog: BE0101)	4 T1 cell B16F10 cell	Hyperthermia significantly increases the proportion of mature DC subtypes (CD11c^+^, CD80^+^, CD86^+^). Hyperthermia stimulates the differentiation of naive T cells to CD8^+^ T cells. Hyperthermia significantly improves the tumor infiltration of CTL. Hyperthermia decreases the levels of CD4, CD25, and Foxp3 in Tregs and reduces the number of MDSC. Hyperthermia increases the expression of IFN‐γ and TNF‐α.	HIT prevented and inhibited the metastasis by forming long‐term immune memory and prolonged survival.
[79]	Photothermal therapy Anti‐PD‐L1 (BMS‐202)	Pan02 cell	Hyperthermia dilates tumor blood vessels and increases the perfusion in TME. Hyperthermia significantly increases the proportion of mature DC subtypes (CD11c^+^, CD80^+^, CD86^+^). Hyperthermia causes more monocytes and DCs to be recruited and infiltrated. Hyperthermia increases the CD8^+^T number in TME. Hyperthermia increases the expression of IFN‐γ, IL‐6, IL‐12, and TNF‐α.	The growth of primary lesions and distant metastases was significantly inhibited in the HIT group.
[82]	Magnetic hyperthermia Anti‐PD‐1 (BE0146, Bio X cell) Anti‐CTLA4 (BE0164, Bio X cell) Radiation therapy	4 T1‐luc cell	The combination therapy increases the number of CD3^+^ T cells.	The combination therapy inhibited the growth of primary lesions and distant metastases but did not significantly prolong survival.

Considerable nanomaterial‐based HIT preclinical studies have also been carried out. Pan et al.^75^ investigated the use of monodisperse superparamagnetic CoFe_2_O_4_@MnFe_2_O_4_ nanoparticles in mouse breast cancer. These nanoparticles exhibited more efficient, stable, and controllable heat energy under an alternating magnetic field. In the HIT group, the number of CD8^+^T cells increased significantly and mortality was the lowest. Hu et al.^76^ verified the HIT efficacy of the mouse CT26 colon tumor model using a copper‐doped nanoscale covalent organic polymer. In the absence of obvious adverse reactions, both primary lesions and metastases were inhibited remarkably in HIT group mice. Furthermore, the survival time of this group was more >50 days, which was better than those of other groups. Luo et al.^77^ carried out HIT by uniting photothermal conversion agents and ICI; they used AA@PN to encapsulate anti‐PD‐1 peptides in a hollow gold nanoshell for photothermal therapy. Comparing single photothermal or ICI monotherapy, the HIT group achieved the best curative effect and the lowest mortality. Distant metastasis models have also been incubated to investigate whether focal photothermal treatment could ameliorate systemic immunoreactivity against cancer and whether it could work in lesions located elsewhere. The metastasis model was impeded markedly by HIT, which affirmed this hypothesis. This finding was accompanied by increases in CD3^+^T, CD4^+^T, and CD8^+^T cells that were one to three times higher than those of the control group on a cytological level. Molecular modulation through secondary immunostimulation, induced under locoregional heating in the TME, has also been shown to significantly enhance TNF‐α, IL‐2, and IFN‐γ, accompanied by a decline in IL‐10.

### 5.2 Mild hyperthermia combined with ICI treatment

Huang et al.^78^ used injectable lipid gel to encapsulate a near‐infrared (NIR) photothermal agent and anti‐PD‐L1. They also controllably released ICIs under a transformation temperature of 39.5°C through NIR heating. Tumors in mice in the HIT group shrunk continuously after irradiation and vanished on the tenth day after therapy. Secular trends in malignancy suppression in the HIT group resulted in the highest 60‐day survival rate (50%). A series of digital information also cytologically revealed that this group had a higher CD8^+^T/CD4^+^T ratio and a higher percentage of mature DC subtypes (CD11c^+^/CD80^+^/CD86^+^). Compared with single anti‐PD‐L1 and MH groups, the CD8^+^T count in the HIT group was 1.5 and 1.4 times higher, respectively. These findings correspond with the findings of Shi et al. regarding surgery and HIT.^73^ Meanwhile, the expressions of CD4^+^, CD25^+^, and FoxP3^+^ on Tregs were significantly lower in the HIT group than in the other groups, as was the number of MDSCs. The above cytological variations provided proof that HIT can relieve intratumoral immune suppression to some extent. Moreover, the abovementioned authors injected carcinoma cells again 30 days after treatment to detect the long‐term immunity to metastases. The HIT group was the only group without metastasis, as it benefited from the establishment of a long‐term adaptive immune response. Yu et al.^79^ encapsulated BMS‐202 (an ICI acting on PD‐L1) in size‐adjustable thermo‐ and fibrotic matrix‐sensitive liposomes. They found that desmoplastic stroma and the intratumoral hypoperfusion of pancreatic carcinoma, which impeded routine treatment, were vanquished by mild hyperthermia combined with ICI treatment. Profiting from the ICI fixed‐point release via NIR, they demonstrated that eutherapeutic remission occurred in both primary lesions and metastasis.

### 5.3 HIT combined with other therapies

Radiotherapy, which is the third kind of major traditional therapy for cancer, has been proved to show strong complementarity in the human body when combined with hyperthermia^80^ or ICI.^81^ Therefore, the exploration of HIT combined with radiotherapy constitutes a promising direction in the field of integrative therapy. HIT was investigated based on multiple therapeutics, including MH, anti‐PD‐L1, anti‐CTLA‐4, and radiotherapy, by Oei et al.^82^ They found that both primary lesions and metastases were constrained in the united group and that T cell infiltration with CD3^+^ recruitment was evident. However, the coherence of therapies did not show any statistical advantage regarding curative effectiveness. It may be that the combinations of heat, radiation, drug dosages, and arrangements used resulted in this unsatisfactory result; it may be that determining the correct arrangement is a complex process, suggesting that future research into a conceptual design is crucial.

Although the accession of eradicative radiotherapy did not provide any survival benefit for mice, research into the combination of HIT with immunomodulatory adjuvants had achieved promising effects. Han et al.^74^ loaded the imiquimod into nanoparticles, with mice in the relevant group achieving a 100% 40‐day survival rate (compared to 0% for other groups). The same experiment also compared the differences in memory T cell generation between each group (surgery, HIFU, surgery with adjuvant, and HIFU with adjuvant). The long‐term, substantial generation of memory T cells was observed in the HIFU with an adjuvant group. Furthermore, the 80‐day survival rate of this group was also the highest (80%), verifying the control of tumor recurrence.

## 6 FORWARD TO CLINICAL ADMINISTRATION

Pioneering studies are now exploring the clinical applications of HIT. A number of studies have achieved preliminary results that are significant. Thus, it is possible to validate and interpret some of the conclusions and considerations arising from these basic experiments. Ongoing HIT clinical trials are summarized in Table 2. Thermal ablation is still the most widely and maturely used form of hyperthermia in clinical practice, with the majority of these studies focusing on hepatocellular carcinoma (HCC). Regarding completed trials, Lyu et al.^83^ performed anti‐PD‐1 (nivolumab + pembrolizumab) combined with thermal ablation in 33 HCC patients who had previously experienced failure during sorafenib therapy. The objective response rate (ORR) increased 2.4 times, and the progression‐free survival (PFS) and median overall survival (mOS) reached 5 and 16.9 months, respectively; these values were higher than those of capecitabine chemotherapy alone (4 and 8 months, respectively).^84^ Duffy et al.^85^ combined tremelimumab (anti‐CTLA‐4) with radiofrequency ablation to access the effects of HIT. They achieved a PFS of 6 months in 57.1% of patients and a 12‐month PFS in 33.1%; they also obtained a time to progression (TTP) of 7.4 months and an OS of 12.3 months. One round of intrahepatic nidus ablation was also observed to lead to the shrinkage of other lesions after the application of anti‐CTLA‐4. This effect may have been related to immune sensitization generated by hyperthermia.

**TABLE 2:** HIT clinical trials registered on clinicaltrials.gov

	Trial Identifier	Recruitment status	Title	Study nature	Participant numbers and diseases	Therapeutic regimens	Primary endpoints	Latest results
1	NCT04220944	Recruiting	Combined Locoregional Treatment With Immunotherapy for Unresectable HCC	Phase I Interventional study Single arm Open label	45 (est) Unresectable HCC	Anti‐PD‐1: Sintilimab (200 mg, per 3 weeks) was administered initially on days 3–7 after the first ablation and TACE. Thermal ablation: The microwave ablation area should cover at least two‐thirds the size of the nodules. **·** TACE: Patient was treated with epirubicin lipiodol emulsion(Epirubicin 40 mg, Lipiodol 10 ml).	PFS	Not yet
2	NCT03939975	Completed	Anti‐PD‐1 therapy Combined With Thermal Ablation for Advanced HCC	Phase II Interventional study Single arm Open label	50 Advanced HCC after sorafenib failure	Anti‐PD‐1: Nivolumab (3 mg/kg, per 2 weeks) or pembrolizumab (2 mg/kg, per 3 weeks) was administered initially on days 3–7 after the first ablation. Thermal ablation: Radiofrequency or microwave ablation was performed with CT guidance. Patients who showed atypical progression 3 months after ablation were repeated.	Safety ORR	The incidence of all grades of irAEs was 82%, with a 14% incidence of severe irAEs. The most frequent AE associated with ablation were pain, vomiting, and transaminitis, which were overall manageable. Severe AE and ablation‐induced ICI discontinuation were not observed. The ORR of HIT was 21.2%, with 6.1% in CR and 15.1% in PR.
3	NCT03867084	Recruiting	Safety and Efficacy of Pembrolizumab (MK‐3475) Versus Placebo as Adjuvant Therapy in Participants With Hepatocellular Carcinoma (HCC) and Complete Radiological Response After Surgical Resection or Local Ablation (MK‐3475‐937 / KEYNOTE‐937)	Phase III Interventional study Randomized allocation Double‐blind	950 (est) HCC after surgical resection or local ablation	Anti‐PD‐1: Pembrolizumab (200 mg, per 3 weeks) was administered intravenously. Randomization needs to be performed within 12 weeks from the date of surgery or ablation. Thermal ablation: Radiofrequency or microwave ablation.	RFS OS	Not yet
4	NCT03864211	Active, not recruiting	Thermal Ablation Followed by Immunotherapy for HCC	Phase I/II Interventional study Randomized allocation Open label	120 (est) Unresectable HCC	Anti‐PD‐1: Toripalimab (240 mg, per 3 weeks) was administered initially on day 3 or day 14 after the ablation. Thermal ablation: Radiofrequency or microwave ablation was performed under CT or ultrasound guidance for one to five target lesions.	PFS	Not yet
5	NCT03753659	Recruiting	IMMULAB ‐ Immunotherapy With Pembrolizumab in Combination With Local Ablation in Hepatocellular Carcinoma (HCC)	Phase II Interventional study Single arm Open label	30 (est) Early stage HCC	Anti‐PD‐1: Pembrolizumab (200 mg, per 3 weeks) was administered intravenously. Ablation/brachytherapy/TACE was performed 2 days prior to the third dose of Pembrolizumab. Thermal ablation: Radiofrequency or microwave ablation was performed via ultrasound‐ or CT‐guided placement of a needle electrode/probe penetrating into the lesion center. Brachytherapy: Brachytherapy was performed via ultrasound‐ or CT‐guided placement of a needle electrode/probe penetrating into the lesion center. TACE: TACE using drug‐eluting beads was performed combined with ablation or brachytherapy	ORR	Not yet
6	NCT04652440	Recruiting	RFA Combined With PD‐1 in HCC: Phase II Study	Phase I/II Interventional study Single arm Open label	30 (est) HCC	Anti‐PD‐1: Tislelizumab was administered intravenously after ablation. Thermal ablation: Radiofrequency ablation.	Safety Tolerability	Not yet
7	NCT04150744	Recruiting	RFA Plus Carrizumab vs Carrizumab Alone for HCC	Phase II Interventional study Non‐Randomized allocation Double‐blind	120 (est) Advanced and unresectable HCC	Anti‐PD‐1: Carrizumab. Thermal ablation: Radiofrequency ablation.	PFS	Not yet
8	NCT01853618	Completed	Tremelimumab With Chemoembolization or Ablation for Liver Cancer	Phase I/II Interventional study Single arm Open label	32 Advanced HCC	Anti‐CTLA‐4: Tremelimumab (3.5 or 10 mg/kg, per 4 weeks) was administered intravenously. Ablation: Radiofrequency or cryotherapy ablation was performed 36 days after initial ICI medication. TACE: Patients with Barcelona Clinic Liver Cancer (BCLC) Stage B received TACE in conjunction with ablation.	Safety Feasibility	There were no significant differences in irAEs between the two doses of Tremelimumab and no dose‐limiting toxicities were encountered. The most common irAEs were pruritus, mainly grade I. The PFS rates at 6 and 12 months were 57.1% and 33.1%, respectively. The TTP was 7.4 months, and the OS was 12.3 months.
9	NCT03337841	Unknown	Pembrolizumab as Neoadjuvant Treatment in HCC	Phase II Interventional study Parallel assignment Open label	50 (est) HCC	Anti‐PD‐1: Pembrolizumab (200 mg) was first administered once before the ablation or surgery and then continued after ablation or surgery(per 3 weeks). Thermal ablation Surgery	One‐year RFS rate	Not yet
10	NCT02821754	Active, not recruiting	A Pilot Study of Combined Immune Checkpoint Inhibition in Combination With Ablative Therapies in Subjects With Hepatocellular Carcinoma (HCC) or Biliary Tract Carcinomas (BTC)	Phase II Interventional study Parallel assignment Open label	54 Advanced HCC Advanced Cholangiocarcinoma	Anti‐CTLA‐4: Tremelimumab (75 mg flat dose, per 4 weeks for 4 doses) was administered intravenously. Anti‐PD‐L1: Durvalumab (1500 mg flat dose, per 4 weeks) was administered intravenously. **A**blation: Radiofrequency or cryotherapy ablation. TACE: Patients with Barcelona Clinic Liver Cancer (BCLC) Stage B received TACE.	PFS	54 patients have been enrolled.
11	NCT03101475	Active, not recruiting	Synergism of Immunomodulation and Tumor Ablation (ILOC)	Phase II Interventional study Single arm Open label	22 (est) Liver metastasis of colorectal cancer	Anti‐CTLA‐4: Tremelimumab (75 mg, per 4 weeks for 4 doses) was administered intravenously. Anti‐PD‐L1: Durvalumab (1500 mg, per 4 weeks) was administered intravenously. **A**blation: Radiofrequency ablation performed percutaneously under CT, MRI, or ultrasound guidance 8 to 14 days after the start of ICI therapy.	Best overall immune response rate	Not yet
12	NCT03393858	Recruiting	Combination of Immunotherapy and Hyperthermia in Advanced Malignant Mesothelioma	Phase I/II Interventional study Single arm Open label	40 (est) Malignant mesothelioma	Anti‐PD‐1: Pembrolizumab (100 mg, per 3 weeks) was administered intravenously. MH: A Thermotron RF‐8EX device was utilized for hyperthermia for 40 min, with the maximum temperature set at 42°C ± 0.5°C as the upper limit, twice a week since the 1st week of Pembrolizumab for a total of 10 times. Dendritic cells‐cytokine induced killer (DC‐CIK) cell immunotherapy: Mononuclear cells were collected from 50 ml peripheral blood and cultured DC‐CIK cells for 15–20 days. Cells were infused back into the patients 3 times via intravenous infusion.	PFS	Not yet
13	NCT03757858	Recruiting	Hyperthermia Combined With Immunotherapy in the Treatment of Abdominal and Pelvic Malignancies or Metastases	Phase I/II Interventional study Non‐Randomized allocation Single‐blind	80 (est) Primary or metastatic carcinoma of abdomen and pelvis	Anti‐PD‐1: Pembrolizumab (100 mg, per 3 weeks) was administered intravenously. MH: A Thermotron RF‐8EX device was utilized for hyperthermia for 40 min, with the maximum temperature set at 42°C ± 0.5°C as the upper limit, twice a week since the 1st week of Pembrolizumab for a total of 10 times. Dendritic cells‐cytokine induced killer (DC‐CIK) cell immunotherapy: Mononuclear cells were collected from 50 mL peripheral blood and cultured DC‐CIK cells for 15–20 days. Cells were infused back into the patients 3 times via intravenous infusion.	Safety ORR	Not yet
14	NCT03959761	Unknown	Tolerance of Intraperitoneal (IP) Nivolumab After Extensive Debulking Surgery and Hyperthermic Intraperitoneal Chemotherapy (HIPEC) in Patients With Advanced Ovarian Carcinoma (ICONIC)	Phase I/II Interventional study Single arm Open label	32 (est) Ovarian cancer after extensive debulking surgery	Anti‐PD‐1: Nivolumab (0.5/1/3 mg/kg, 4 times per 2 weeks) was administered via an intraperitoneal catheter on days 5–7 after surgery and HIPEC. Hyperthermic intraperitoneal chemotherapy Extensive debulking surgery	Safety	Not yet
15	NCT04889768	Not yet recruiting	HIPEC Combined With Camrelizumab, Paclitaxel, and S‐1 for Conversion Therapy in Patients With Advanced Gastric Cancer With Peritoneal Metastasis	Interventional study Single arm Open label	46 (est) Gastric cancer with peritoneal metastasis	Anti‐PD‐1: Camrelizumab (200 mg, per 3 weeks) was administered intravenously. Hyperthermic intraperitoneal chemotherapy: Taxol (Paclitaxel Injection) was administered via an intraperitoneal catheter (75 mg/m,^2^ d1, d3). Chemotherapy: Taxol (150 mg/m,^2^ d1, per 3 weeks) was administered intravenously. S‐1 (80‐120 mg/m,^2^ d1‐14, per 3 weeks) was orally administered. Surgical: Through the above treatment, if the peritoneal carcinomatosis index (PCI) is less than 20, then consider the cytoreductive surgery (resection of primary tumors and metastases). For inoperable patients, continue to the above treatment.	The rate of R0 resection	Not yet
16	NCT04156087	Recruiting	Progression‐free Survival After MWA Plus Durvalumab and Tremelimumab for Unresectable Locally Advanced Pancreatic Cancer (MIMIPAC)	Phase II Interventional study Single arm Open label	20 (est) Unresectable Non‐metastatic Locally Advanced Pancreatic Cancer	Anti‐CTLA‐4: Tremelimumab (75 mg, per 4 weeks for 4 doses) was administered intravenously. Anti‐PD‐L1: Durvalumab (1500 mg, per 4 weeks) was administered intravenously. Ablation: Minimally invasive surgical microwave ablation (MIS‐MWA) of the pancreatic tumor was performed two weeks after the first dose of Durvalumab and Tremelimumab. Chemotherapy: Systemic gemcitabine (1000 mg /m^2^) was started 6 weeks after MIS‐MWA and given once a week for 3 weeks, followed by a week of rest.	PFS	Not yet
17	NCT04805736	Recruiting	Microwave Ablation Combined With Camrelizumab in the Treatment of Early Breast Cancer	Phase II Interventional study Non‐Randomized allocation Open label	60 (est) Early newly diagnosed breast cancer	Anti‐PD‐1: Camrelizumab (200 mg, per 3 weeks) was administered intravenously a few days after microwave ablation. Thermal ablation: Microwave ablation was performed with image guidance 7–10 days prior to surgery. Surgery: Standard of care breast‐conserving surgery or radical mastectomy.	Safety	Not yet
18	NCT03237572	Recruiting	Focused Ultrasound and Pembrolizumab in Metastatic Breast Cancer (Breast‐48)	Phase I Interventional study Randomized allocation Open label	15 (est) Breast cancer	Anti‐PD‐1: Pembrolizumab (200 mg, per 3 weeks) was administered intravenously. Thermal ablation: High‐intensity focused ultrasound (HIFU) ablation was target 50% of the tumor, up to 3 cubic centimeters. HIFU was performed 2 weeks after the initial ICI medication.	Change of TIL	Not yet
19	NCT02469701	Terminated	Advanced Non‐Small Cell Lung Cancer Progressing After at Least One Prior Therapy For Metastatic Disease	Phase II Interventional study Single arm Open label	10 Advanced NSCLC with metastasis	Anti‐PD‐1: Nivolumab (3 mg/kg, per 2 weeks) was administered intravenously. Ablation: Thermal ablation or cryotherapy.	Safety	Enrollment of the full expected number of patients was completed. The total AE incidence was 100%, most commonly pain (100%), anemia (80%), anorexia (60%), and fatigue (60%). Severe AEs were 50% and all‐cause mortality was 0%.
20	NCT04116320	Recruiting	Focused Ultrasound Ablation and PD‐1 Antibody Blockade in Advanced Solid Tumors (AM‐003)	Phase I Interventional study Non‐Randomized allocation Open label	32 (est) Advanced solid tumors	Anti‐PD‐1 Thermal ablation: The Echopulse device delivers focused ultrasound ablation (FUSA) therapy. Adjuvant: Imiquimod as a TLR7 agonist therapy.	Safety Toxicity CD8^+^ T cell infiltration	Not yet

Besides HCC, Xie et al.^86^ applied tremelimumab in HIT to treat biliary tract cancer. Even though 80% of the participants had progressed after second‐line chemotherapy, the PFS was still longer than that obtained in a large retrospective analysis of second‐line chemotherapy (3.4 months vs. 2.8 months).^87^ In addition, only 10% of the patients observed grade 3 or 4 irAEs. Kleef et al.^88^ studied stage IV triple‐negative breast cancer patients with lung metastases; these patients received HIT therapy comprising 13.56 Hz of local radiofrequency and IL‐2 induced general hyperthermia, combined with immunotherapy (nivolumab+ipilimumab). As a result, the Karnofsky performance score increased from 80 to 100, the pulmonary metastasis was a complete response (CR), and a final survival time of 27 months was reached. Based on this research, Kleef et al.^89^ also performed a retrospective study of 131 patients with different types of cancer. The immunotherapy plan comprised ipilimumab (0.3 mg·kg^−1^) plus nivolumab (0.5 mg·kg^−1^), and cyclophosphamide was used to decrease the number of Tregs. For hyperthermia, based on the above‐mentioned radiofrequency and IL‐2, water filter infrared whole‐body hyperthermia was performed. Finally, an ORR of 31.3% and a PFS of 10 months were attained. Wei et al.^90^ conducted a non‐small cell lung cancer (NSCLC) perspective study involving 21 patients who could not undergo surgery and targeted therapy, to assess the effects of HIT. Camrelizumab (200 mg, q2w) was infused from the 5th to 7th days after microwave ablation until progression. Eventually, an ORR of 33.3% was obtained, two patients achieved CR, five achieved PR, and a mOS of 5.1 months was reached.

Although the above results have inspired HIT to be incorporated into clinical practice, more large‐scale clinical trials are needed to better guide the current understanding of the thermal dose, adverse reactions, and applicable population. Matsumoto et al.^91^ revealed that the maturation of DCs occurred when both DCs and tumors received continuous thermostimulation. This may mainly be attributed to HIT sensitization being affected by intricate multicellular and polymolecular cascades. Chen et al.^92^ found that sequential hyperthermia could reduce the density of Tregs in TME, but that single hyperthermia could not. Incomplete ablation has also been reported to promote cancer metastasis, but expanding the ablation range will inevitably cause secondary death damage to surrounding normal tissues.^93^ Therefore, it is crucial to optimize the temperature and scope regarding HIT. Studies have also addressed the adverse reactions of HIT, which should be analyzed through the effect of hyperthermia on irAEs. Regarding the application of anti‐CTLA‐4, the toxicity of the gastrointestinal tract, skin, and pituitary should be monitored.^94^ The adverse reactions of the lung, thyroid, and myasthenia gravis should also be scrutinized when using anti‐PD‐1/PD‐L1.^95^ ICIs have been associated with myocarditis and more severe mortality in multiple ICI treatment groups (0.27%:0.06%, *p* < 0.001), suggesting that attention should also be paid to this issue regarding HIT.^96^ Kleef found that only 23.66, 16.03, 6.11, and 2.29% of patients observed irAEs of grades 1, 2, 3, and 4, respectively.^89^ This indicates that hyperthermia could potentially ameliorate irAEs. This may arise from the lower drug doses that can be used under sensibilization. However, hyperthermia can change the distribution of circulating blood volume by regulating blood vessels. It is not clear whether this will affect the occurrence of ICI‐related myocarditis; follow‐up research should address this question.

Any therapy transition from the lab toward the ward is gradually step‐by‐step, and the same is true for HIT as an emerging discipline. As of now, the implementation of hyperthermia is still far less than the demand. The cost‐effectiveness of HIT is a considerable factor influencing the psychological acceptance of patients. For UH, several studies have shown that thermal ablation exhibit superior cost‐effectiveness compared to surgery.^97^, ^98^ Although MH is primarily used as an adjunct to treatment as an expansion option, its addition does not result in lower cost‐effectiveness. Trevor D Hamilton's study showed that the addition of HIPEC had better cost‐effectiveness than systemic chemotherapy alone for cancer with peritoneal metastases.^99^ Considering the above views, it can be inferred that the addition of hyperthermia could increase the cost‐effectiveness of ICI, which effectively improves psychological acceptance. On the other hand, for patients with a limited understanding of hyperthermia, it is crucial to do well doctor‐patient communication to eliminate patients' doubts and to explain and handle AEs properly. At present, there is still potential for further expansion of HIT indications. Under the premise of excluding contraindications such as thermanesthesia, bleeding tendency, unstable vital signs, and severe cardiopulmonary dysfunction, patients receiving ICI are widely administrable candidates for extracorporeal thermogenic MH (e.g., whole‐body hyperthermia, local external irradiation). To determine whether patients with pleural or peritoneal metastases can be treated with intracavity hyperthermia chemotherapy based on ICI, the patient's tolerance to chemotherapy should also be taken into account. Meanwhile, patients with the propensity for bleeding and infection should be strictly limited to intracavity hyperthermia chemotherapy due to the invasiveness. Regarding the radicalness of UH, the indication HIT involving UH should focus on the reference of whether patients are suitable for ablation. After clearly meeting ablation indications, the application of ICI should consider the condition and the curative biomarker, such as advanced multiple metastases, and molecular subtypes identified as PD‐L1 positive or dMMR/MSIH, which can be systemic administration of ICI after hyperthermia. Overall, it makes sense to enrich the availability of HIT, and it deserves to be more actively incorporated into comprehensive treatment based on the acceptance of patients. Medical institutions should also strengthen training on quality supervision and standardization of hyperthermia, and add hyperthermia equipment to provide more therapeutic options and better hardware bases for physicians and patients.

## 7 OUTLOOK

Many outcomes from human body studies will be published in the near future; it is expected that these results will provide causes for optimism. Nevertheless, more in vitro studies are needed to reduce the opportunity costs of clinical program selection. The future progression of HIT relies on the development of hyperthermia and ICIs. To exploit potential ICs, it is essential to explore new inhibitors, define ICI biomarkers, improve ICI efficacy, and reduce the side effects; these efforts will help to realize this promising avenue for future cancer immunology.^100^ For hyperthermia, meanwhile, major directions for future research will include real‐time thermometry, precise thermal control, and exact heating.^101^ Magnetic resonance (MR), computed tomography (CT), and ultrasonography (US)‐based three‐dimensional (3D) non‐invasive thermometry techniques are developing rapidly.^102^ The advanced infrared real‐time thermometry instrument can also conduct two‐dimensional (2D) thermography via energy distribution detection.^103^ The above technologies provide a better platform for quality control regarding hyperthermia.

By virtue of their special physicochemical properties, nanomaterials can exert many superior effects. They may gradually lead to a breakthrough for precise hyperthermia in the future. Nanomaterial‐based drug delivery platforms are deemed to represent a new model of “targeted therapy,” which can optimize pharmacokinetics, improve bioavailability, and reduce toxicity. DAMP triggered by hyperthermia offers a recognizable targeting platform for nanoparticles with encapsulated drugs. This could become a breakthrough point for exact HIT as a form of next‐generation target therapy. Optimizing conventional pharmacokinetics could augment bioavailability and abate the toxicity of ICIs. By utilizing the up‐regulated expression of HSP70 protein under heating, a precise photothermal remedy platform has been developed and a eutherapeutic outcome has been achieved in mice.^104^ Mirroring this, there are plans to further develop nano‐targeted photothermal therapy while loading ICI. To date, nanomedicine has been extensively utilized in HIT, such as in ICI‐encapsulated controlled‐release patches, thermosensitive drug‐carrying gel, photothermal sensitizers, and nano‐adjuvants.^74^, ^105^, ^106^ These applications have shown exciting results and promise an exciting future regarding nano‐oncology.

A heterojunction WO_2.9_‐WSe_2_‐PEG nanoradiosensitizer constructed by Dong et al.^107^ has realized in vivo radiotherapy, hyperthermia, and immunotherapy. This achievement can build a bridge for HIT‐based combination therapy. These practices will aid the exploration of wider combination remedies, such as targeting, surgery, and chemotherapy on multiple levels.

## 8 CONCLUSIONS

Based on the limitations of monotherapy regarding advanced cancer, and building on the verification of the multifactorial influences of hyperthermia on tumor immunization, it is necessary to continue to explore HIT, and to promote deeper and broader investigations. Several HIT sensitization mechanisms have not only been observed and explained in numerous basic experiments but have also been reflected and demonstrated as significant survival benefits for patients through pioneering clinical trials. Further studies into the theoretical basis and practical standards of HIT, combined with larger‐scale clinical studies involving more cancer types, will be necessary for the future.

## AUTHOR CONTRIBUTION

Pengyuan Liu, Mengna Ye, and Yajun Wu contributed equally to this review and should be considered the co‐first author. Conceptualizations, Pengyuan Liu and Zhibing Wu; literature collecting and preparation, Mengna Ye and Yajun Wu; writing‐original draft preparation, Pengyuan Liu; writing‐review and editing, Pengyuan Liu, Mengna Ye, Yajun Wu, Lichao Wu, and Kaiping Lan; Supervision, Zhibing Wu, Kaiping Lan.

## CONFLICT OF INTEREST

The authors declare that they have no competing interests. This is an open access article under the terms of the Creative Commons Attribution License, which permits use, distribution, and reproduction in any medium provided the original work is properly cited.

## ETHICS APPROVAL

This study was approved by the Ethics Department of Zhejiang Hospital.

## Data Availability

The data used to support the findings of this study are available from the corresponding author upon request.

## References

[1] Welch HG , Kramer BS , Black WC . Epidemiologic signatures in cancer. N Engl J Med. 2019;381(14):1378‐1386. doi:10.1056/NEJMsr1905447 31577882

[2] Wild CP , Weiderpass E , Stewart BW . World Cancer Report: Cancer Research For Cancer Prevention. IRAC; 2020. 39432694

[3] Siegel RL , Miller KD , Jemal A . Cancer statistics, 2020. CA Cancer J Clin. 2020;70(1):7‐30. doi:10.3322/caac.21590 31912902

[4] Bockamp E , Rosigkeit S , Siegl D , Schuppan D . Nano‐enhanced cancer immunotherapy: immunology encounters nanotechnology. Cells. 2020;9(9):2102. Published 2020 Sep 15. doi:10.3390/cells9092102 32942725 PMC7565449

[5] Sanmamed MF , Chen L . A paradigm shift in cancer immunotherapy: from enhancement to normalization [published correction appears in cell. 2019 Jan 24;176(3):677]. Cell. 2018;175(2):313‐326. doi:10.1016/j.cell.2018.09.035 30290139 PMC6538253

[6] Waldman AD , Fritz JM , Lenardo MJ . A guide to cancer immunotherapy: from T cell basic science to clinical practice. Nat Rev Immunol. 2020;20(11):651‐668. doi:10.1038/s41577-020-0306-5 32433532 PMC7238960

[7] Johdi NA , Sukor NF . Colorectal cancer immunotherapy: options and strategies. Front Immunol. 2020;11:1624. doi:10.3389/fimmu.2020.01624 33042104 PMC7530194

[8] Galluzzi L , Humeau J , Buqué A , Zitvogel L , Kroemer G . Immunostimulation with chemotherapy in the era of immune checkpoint inhibitors. Nat Rev Clin Oncol. 2020;17(12):725‐741. doi:10.1038/s41571-020-0413-z 32760014

[9] Hurwitz MD . Hyperthermia and immunotherapy: clinical opportunities. Int J Hyperthermia. 2019;36(sup1):4‐9. doi:10.1080/02656736.2019.1653499 31795827

[10] Eroglu Z , Zaretsky JM , Hu‐Lieskovan S , et al. High response rate to PD‐1 blockade in desmoplastic melanomas. Nature. 2018;553(7688):347‐350. doi:10.1038/nature25187 29320474 PMC5773412

[11] Le DT , Durham JN , Smith KN , et al. Mismatch repair deficiency predicts response of solid tumors to PD‐1 blockade. Science. 2017;357(6349):409‐413. doi:10.1126/science.aan6733 28596308 PMC5576142

[12] Cristescu R , Mogg R , Ayers M , et al. Pan‐tumor genomic biomarkers for PD‐1 checkpoint blockade‐based immunotherapy [published correction appears in Science. 2019 Mar 1;363(6430)]. Science. 2018;362(6411):eaar3593. doi:10.1126/science.aar3593 30309915 PMC6718162

[13] Garon EB , Rizvi NA , Hui R , et al. Pembrolizumab for the treatment of non‐small‐cell lung cancer. N Engl J Med. 2015;372(21):2018‐2028. doi:10.1056/NEJMoa1501824 25891174

[14] Ferris RL , Blumenschein G Jr , Fayette J , et al. Nivolumab for recurrent squamous‐cell carcinoma of the head and neck. N Engl J Med. 2016;375(19):1856‐1867. doi:10.1056/NEJMoa1602252 27718784 PMC5564292

[15] Rosenberg JE , Hoffman‐Censits J , Powles T , et al. Atezolizumab in patients with locally advanced and metastatic urothelial carcinoma who have progressed following treatment with platinum‐based chemotherapy: a single‐arm, multicentre, phase 2 trial. Lancet. 2016;387(10031):1909‐1920. doi:10.1016/S0140-6736(16)00561-4 26952546 PMC5480242

[16] Bellmunt J , de Wit R , Vaughn DJ , et al. Pembrolizumab as second‐line therapy for advanced urothelial carcinoma. N Engl J Med. 2017;376(11):1015‐1026. doi:10.1056/NEJMoa1613683 28212060 PMC5635424

[17] Zhou B , Gao Y , Zhang P , Chu Q . Acquired resistance to immune checkpoint blockades: the underlying mechanisms and potential strategies. Front Immunol. 2021;12:693609. doi:10.3389/fimmu.2021.693609 34194441 PMC8236848

[18] Gotwals P , Cameron S , Cipolletta D , et al. Prospects for combining targeted and conventional cancer therapy with immunotherapy. Nat Rev Cancer. 2017;17(5):286‐301. doi:10.1038/nrc.2017.17 28338065

[19] Singh D . Current updates and future perspectives on the management of renal cell carcinoma. Life Sci. 2021;264:118632. doi:10.1016/j.lfs.2020.118632 33115605

[20] Yu JH , Wang ZZ , Fan YC , et al. Comparison of neoadjuvant chemotherapy followed by surgery vs. surgery alone for locally advanced gastric cancer: a meta‐analysis. Chin Med J (Engl). 2021;134(14):1669‐1680. doi:10.1097/CM9.0000000000001603 34397593 PMC8318625

[21] Winer LK , Cortez AR , Ahmad SA , et al. Evaluating the impact of ESPAC‐1 on shifting the paradigm of pancreatic cancer treatment. J Surg Res. 2021;259:442‐450. doi:10.1016/j.jss.2020.09.009 33059910

[22] Wang C , Lu X , Lyu Z , Bi N , Wang L . Comparison of up‐front radiotherapy and TKI with TKI alone for NSCLC with brain metastases and EGFR mutation: a meta‐analysis. Lung Cancer. 2018;122:94‐99. doi:10.1016/j.lungcan.2018.05.014 30032853

[23] Stelmachowska‐Banaś M , Czajka‐Oraniec I . Management of endocrine immune‐related adverse events of immune checkpoint inhibitors: an updated review. Endocr Connect. 2020;9(10):R207‐R228. doi:10.1530/EC-20-0342 33064663 PMC7576644

[24] Sha CM , Lehrer EJ , Hwang C , et al. Toxicity in combination immune checkpoint inhibitor and radiation therapy: a systematic review and meta‐analysis. Radiother Oncol. 2020;151:141‐148. doi:10.1016/j.radonc.2020.07.035 32717359

[25] Foster J , Hodder SG , Lloyd AB , Havenith G . Individual responses to heat stress: implications for hyperthermia and physical work capacity. Front Physiol. 2020;11:541483. doi:10.3389/fphys.2020.541483 33013476 PMC7516259

[26] Amin M , Huang W , Seynhaeve ALB , Ten Hagen TLM . Hyperthermia and temperature‐sensitive nanomaterials for spatiotemporal drug delivery to solid tumors. Pharmaceutics. 2020;12(11):1007. doi:10.3390/pharmaceutics12111007 33105816 PMC7690578

[27] Dellinger TH , Han ES . State of the science: the role of HIPEC in the treatment of ovarian cancer. Gynecol Oncol. 2021;160(2):364‐368. doi:10.1016/j.ygyno.2020.12.029 33419611

[28] Tan W , Deng Q , Lin S , Wang Y , Xu G . Comparison of microwave ablation and radiofrequency ablation for hepatocellular carcinoma: a systematic review and meta‐analysis. Int J Hyperthermia. 2019;36(1):264‐272. doi:10.1080/02656736.2018.1562571 30676100

[29] Morita M , Kuwano H , Araki K , et al. Prognostic significance of lymphocyte infiltration following preoperative chemoradiotherapy and hyperthermia for esophageal cancer. Int J Radiat Oncol Biol Phys. 2001;49(5):1259‐1266. doi:10.1016/s0360-3016(00)01465-6 11286832

[30] Cheng HW , Tsao HY , Chiang CS , Chen SY . Advances in magnetic nanoparticle‐mediated cancer immune‐theranostics. Adv Healthc Mater. 2021;10(1):e2001451. doi:10.1002/adhm.202001451 33135398

[31] Duan X , Wang M , Han X , et al. Combined use of microwave ablation and cell immunotherapy induces nonspecific immunity of hepatocellular carcinoma model mice. Cell Cycle. 2020;19(24):3595‐3607. doi:10.1080/15384101.2020.1853942 33283623 PMC7781627

[32] Kole C , Charalampakis N , Tsakatikas S , et al. Immunotherapy for Hepatocellular Carcinoma: A 2021 Update. Cancers (Basel). 2020;12(10):2859. doi:10.3390/cancers12102859 33020428 PMC7600093

[33] Rangamuwa K , Leong T , Weeden C , et al. Thermal ablation in non‐small cell lung cancer: a review of treatment modalities and the evidence for combination with immune checkpoint inhibitors. Transl Lung Cancer Res. 2021;10(6):2842‐2857. doi:10.21037/tlcr-20-1075 34295682 PMC8264311

[34] Li Z , Deng J , Sun J , Ma Y . Hyperthermia targeting the tumor microenvironment facilitates immune checkpoint inhibitors. Front Immunol. 2020;11:595207. doi:10.3389/fimmu.2020.595207 33240283 PMC7680736

[35] Chang M , Hou Z , Wang M , Li C , Lin J . Recent advances in hyperthermia therapy‐based synergistic immunotherapy. Adv Mater. 2021;33(4):e2004788. doi:10.1002/adma.202004788 33289219

[36] Tang S , Ning Q , Yang L , Mo Z , Tang S . Mechanisms of immune escape in the cancer immune cycle. Int Immunopharmacol. 2020;86:106700. doi:10.1016/j.intimp.2020.106700 32590316

[37] Dong Y , Wong JSL , Sugimura R , et al. Recent advances and future prospects in immune checkpoint (ICI)‐based combination therapy for advanced HCC. Cancers (Basel). 2021;13(8):1949. Published 2021 Apr 18. doi:10.3390/cancers13081949 33919570 PMC8072916

[38] Das S , Johnson DB . Immune‐related adverse events and anti‐tumor efficacy of immune checkpoint inhibitors. J Immunother Cancer. 2019;7(1):306. doi:10.1186/s40425-019-0805-8 31730012 PMC6858629

[39] Ho WJ , Jaffee EM , Zheng L . The tumour microenvironment in pancreatic cancer ‐ clinical challenges and opportunities. Nat Rev Clin Oncol. 2020;17(9):527‐540. doi:10.1038/s41571-020-0363-5 32398706 PMC7442729

[40] Klemm F , Joyce JA . Microenvironmental regulation of therapeutic response in cancer. Trends Cell Biol. 2015;25(4):198‐213. doi:10.1016/j.tcb.2014.11.006 25540894 PMC5424264

[41] Wang S , He Z , Wang X , Li H , Liu XS . Antigen presentation and tumor immunogenicity in cancer immunotherapy response prediction. Elife. 2019;8:e49020. doi:10.7554/eLife.49020 31767055 PMC6879305

[42] Goliwas KF , Deshane JS , Elmets CA , Athar M . Moving immune therapy Forward targeting TME. Physiol Rev. 2021;101(2):417‐425. doi:10.1152/physrev.00008.2020 32790578 PMC8428923

[43] Lee SY , Fiorentini G , Szasz AM , Szigeti G , Szasz A , Minnaar CA . Quo vadis oncological hyperthermia (2020)? Front Oncol. 2020;10:1690. doi:10.3389/fonc.2020.01690 33014841 PMC7499808

[44] Cheng Y , Weng S , Yu L , Zhu N , Yang M , Yuan Y . The role of hyperthermia in the multidisciplinary treatment of malignant tumors. Integr Cancer Ther. 2019;18:1534735419876345. doi:10.1177/1534735419876345 31522574 PMC7242805

[45] Payne M , Bossmann SH , Basel MT . Direct treatment versus indirect: thermo‐ablative and mild hyperthermia effects. Wiley Interdiscip Rev Nanomed Nanobiotechnol. 2020;12(5):e1638. doi:10.1002/wnan.1638 32352660

[46] De Vita E , De Landro M , Massaroni C , et al. Fiber optic sensors‐based thermal analysis of perfusion‐mediated tissue cooling in liver undergoing laser ablation. IEEE Trans Biomed Eng. 2021;68(3):1066‐1073. doi:10.1109/TBME.2020.3004983 32746040

[47] Scutigliani EM , Liang Y , Crezee H , Kanaar R , Krawczyk PM . Modulating the heat stress response to improve hyperthermia‐based anticancer treatments. Cancers (Basel). 2021;13(6):1243. Published 2021 Mar 12. doi:10.3390/cancers13061243 33808973 PMC8001574

[48] Ng KKC , Chok KSH , Chan ACY , et al. Randomized clinical trial of hepatic resection versus radiofrequency ablation for early‐stage hepatocellular carcinoma. Br J Surg. 2017;104(13):1775‐1784. doi:10.1002/bjs.10677 29091283

[49] van Rhoon GC , Franckena M , Ten Hagen TLM . A moderate thermal dose is sufficient for effective free and TSL based thermochemotherapy. Adv Drug Deliv Rev. 2020;163‐164:145‐156. doi:10.1016/j.addr.2020.03.006 32247801

[50] Mortezaee K , Najafi M . Immune system in cancer radiotherapy: resistance mechanisms and therapy perspectives. Crit Rev Oncol Hematol. 2021;157:103180. doi:10.1016/j.critrevonc.2020.103180 33264717

[51] Liu X , Hogg GD , DeNardo DG . Rethinking immune checkpoint blockade: ‘Beyond the T cell’. J Immunother Cancer. 2021;9(1):e001460. doi:10.1136/jitc-2020-001460 33468555 PMC7817791

[52] Lv W , Cao M , Liu J , Hei Y , Bai J . Tumor microenvironment‐responsive nanozymes achieve photothermal‐enhanced multiple catalysis against tumor hypoxia [published online ahead of print, 2021 Aug 15]. Acta Biomater. 2021;135:617‐627. doi:10.1016/j.actbio.2021.08.015 34407474

[53] Liu Q , Fan T , Zheng Y , et al. Immunogenic exosome‐encapsulated black phosphorus nanoparticles as an effective anticancer photo‐nanovaccine. Nanoscale. 2020;12(38):19939‐19952. doi:10.1039/d0nr05953f 32991664

[54] Toraya‐Brown S , Fiering S . Local tumour hyperthermia as immunotherapy for metastatic cancer. Int J Hyperthermia. 2014;30(8):531‐539. doi:10.3109/02656736.2014.968640 25430985 PMC4558619

[55] Dieing A , Ahlers O , Hildebrandt B , et al. The effect of induced hyperthermia on the immune system. Prog Brain Res. 2007;162:137‐152. doi:10.1016/S0079-6123(06)62008-6 17645918

[56] Du G , Liu Y , Li J , Liu W , Wang Y , Li H . Hypothermic microenvironment plays a key role in tumor immune subversion. Int Immunopharmacol. 2013;17(2):245‐253. doi:10.1016/j.intimp.2013.06.018 23831011

[57] Guo D , Chen Y , Wang S , et al. Exosomes from heat‐stressed tumour cells inhibit tumour growth by converting regulatory T cells to Th17 cells via IL‐6. Immunology. 2018;154(1):132‐143. doi:10.1111/imm.12874 29197065 PMC5904701

[58] Mace TA , Zhong L , Kokolus KM , Repasky EA . Effector CD8+ T cell IFN‐γ production and cytotoxicity are enhanced by mild hyperthermia. Int J Hyperthermia. 2012;28(1):9‐18. doi:10.3109/02656736.2011.616182 22235780 PMC3293214

[59] Cippitelli M , Fionda C , Di Bona D , Piccoli M , Frati L , Santoni A . Hyperthermia enhances CD95‐ligand gene expression in T lymphocytes. J Immunol. 2005;174(1):223‐232. doi:10.4049/jimmunol.174.1.223 15611244

[60] Hou YJ , Yang XX , Liu RQ , et al. Pathological mechanism of photodynamic therapy and photothermal therapy based on nanoparticles. Int J Nanomedicine. 2020;15:6827‐6838. doi:10.2147/IJN.S269321 32982235 PMC7501968

[61] Ahmed A , Tait SWG . Targeting immunogenic cell death in cancer. Mol Oncol. 2020;14(12):2994‐3006. doi:10.1002/1878-0261.12851 33179413 PMC7718954

[62] He K , Liu P , Xu LX . The cryo‐thermal therapy eradicated melanoma in mice by eliciting CD4+ T‐cell‐mediated antitumor memory immune response. Cell Death Dis. 2017;8(3):e2703. Published 2017 Mar 23. doi:10.1038/cddis.2017.125 28333145 PMC5386530

[63] Xue T , Liu P , Zhou Y , et al. Interleukin‐6 induced "acute" phenotypic microenvironment promotes Th1 anti‐tumor immunity in Cryo‐thermal therapy revealed by shotgun and parallel reaction monitoring proteomics. Theranostics. 2016;6(6):773‐794. doi:10.7150/thno.14394 27162549 PMC4860887

[64] Osipov A , Lim SJ , Popovic A , et al. Tumor mutational burden, toxicity, and response of immune checkpoint inhibitors targeting PD(L)1, CTLA‐4, and combination: a meta‐regression analysis. Clin Cancer Res. 2020;26(18):4842‐4851. doi:10.1158/1078-0432.CCR-20-0458 32586938 PMC7501151

[65] Joiner JB , Pylayeva‐Gupta Y , Dayton PA . Focused ultrasound for immunomodulation of the tumor microenvironment. J Immunol. 2020;205(9):2327‐2341. doi:10.4049/jimmunol.1901430 33077668 PMC7583653

[66] Rangamuwa K , Leong T , Bozinovski S , et al. Increase in tumour PD‐L1 expression in non‐small cell lung cancer following bronchoscopic thermal vapour ablation. Transl Lung Cancer Res. 2021;10(6):2858‐2864. doi:10.21037/tlcr-21-76 34295683 PMC8264342

[67] Wu Y , Li Q , Shim G , Oh YK . Melanin‐loaded CpG DNA hydrogel for modulation of tumor immune microenvironment. J Control Release. 2021;330:540‐553. doi:10.1016/j.jconrel.2020.12.040 33373649

[68] Adnan A , Muñoz NM , Prakash P , Habibollahi P , Cressman ENK , Sheth RA . Hyperthermia and tumor immunity. Cancers (Basel). 2021;13(11):2507. Published 2021 May 21. doi:10.3390/cancers13112507 34063752 PMC8196672

[69] Garg AD , Romano E , Rufo N , Agostinis P . Immunogenic versus tolerogenic phagocytosis during anticancer therapy: mechanisms and clinical translation. Cell Death Differ. 2016;23(6):938‐951. doi:10.1038/cdd.2016.5 26891691 PMC4987738

[70] Cao G , Wang J , Zheng X , Wei H , Tian Z , Sun R . Tumor therapeutics work as stress inducers to enhance tumor sensitivity to natural killer (NK) cell cytolysis by up‐regulating NKp30 ligand B7‐H6. J Biol Chem. 2015;290(50):29964‐29973. doi:10.1074/jbc.M115.674010 26472927 PMC4705966

[71] Kubista B , Trieb K , Blahovec H , Kotz R , Micksche M . Hyperthermia increases the susceptibility of chondro‐ and osteosarcoma cells to natural killer cell‐mediated lysis. Anticancer Res. 2002;22(2A):789‐792. 12014651

[72] van den Bijgaart RJ , Eikelenboom DC , Hoogenboom M , Fütterer JJ , den Brok MH , Adema GJ . Thermal and mechanical high‐intensity focused ultrasound: perspectives on tumor ablation, immune effects and combination strategies. Cancer Immunol Immunother. 2017;66(2):247‐258. doi:10.1007/s00262-016-1891-9 27585790 PMC5281669

[73] Shi L , Chen L , Wu C , et al. PD‐1 blockade boosts radiofrequency ablation‐elicited adaptive immune responses against tumor. Clin Cancer Res. 2016;22(5):1173‐1184. doi:10.1158/1078-0432.CCR-15-1352 26933175 PMC4780056

[74] Han X , Wang R , Xu J , et al. In situ thermal ablation of tumors in combination with nano‐adjuvant and immune checkpoint blockade to inhibit cancer metastasis and recurrence. Biomaterials. 2019;224:119490. doi:10.1016/j.biomaterials.2019.119490 31542515

[75] Pan J , Hu P , Guo Y , et al. Combined magnetic hyperthermia and immune therapy for primary and metastatic tumor treatments. ACS Nano. 2020;14(1):1033‐1044. doi:10.1021/acsnano.9b08550 31935064

[76] Hu C , Cai L , Liu S , Liu Y , Zhou Y , Pang M . Copper‐doped nanoscale covalent organic polymer for augmented photo/Chemodynamic synergistic therapy and immunotherapy. Bioconjug Chem. 2020;31(6):1661‐1670. doi:10.1021/acs.bioconjchem.0c00209 32393025

[77] Luo L , Yang J , Zhu C , et al. Sustained release of anti‐PD‐1 peptide for perdurable immunotherapy together with photothermal ablation against primary and distant tumors. J Control Release. 2018;278:87‐99. doi:10.1016/j.jconrel.2018.04.002 29626502

[78] Huang L , Li Y , Du Y , et al. Mild photothermal therapy potentiates anti‐PD‐L1 treatment for immunologically cold tumors via an all‐in‐one and all‐in‐control strategy. Nat Commun. 2019;10(1):4871. doi:10.1038/s41467-019-12771-9 31653838 PMC6814770

[79] Yu Q , Tang X , Zhao W , et al. Mild hyperthermia promotes immune checkpoint blockade‐based immunotherapy against metastatic pancreatic cancer using size‐adjustable nanoparticles☆ [published online ahead of print, 2021 may 14]. Acta Biomater. 2021;133:244‐256. doi:10.1016/j.actbio.2021.05.002 34000465

[80] Lloret M , García‐Cabrera L , Hernandez A , Santana N , López‐Molina L , Lara PC . Feasibility of a deep hyperthermia and radiotherapy programme for advanced tumors: first Spanish experience. Clin Transl Oncol. 2019;21(12):1771‐1775. doi:10.1007/s12094-019-02097-9 31102061

[81] Li W , Yu H . Separating or combining immune checkpoint inhibitors (ICIs) and radiotherapy in the treatment of NSCLC brain metastases. J Cancer Res Clin Oncol. 2020;146(1):137‐152. doi:10.1007/s00432-019-03094-9 31813004 PMC11804339

[82] Oei AL , Korangath P , Mulka K , et al. Enhancing the abscopal effect of radiation and immune checkpoint inhibitor therapies with magnetic nanoparticle hyperthermia in a model of metastatic breast cancer. Int J Hyperthermia. 2019;36(sup1):47‐63. doi:10.1080/02656736.2019.1685686 31795835 PMC7017719

[83] Lyu N , Kong Y , Li X , et al. Ablation reboots the response in advanced hepatocellular carcinoma with stable or atypical response during PD‐1 therapy: a proof‐of‐concept study. Front Oncol. 2020;10:580241. doi:10.3389/fonc.2020.580241 33163408 PMC7581675

[84] Granito A , Marinelli S , Terzi E , et al. Metronomic capecitabine as second‐line treatment in hepatocellular carcinoma after sorafenib failure. Dig Liver Dis. 2015;47(6):518‐522. doi:10.1016/j.dld.2015.03.010 25861840

[85] Duffy AG , Ulahannan SV , Makorova‐Rusher O , et al. Tremelimumab in combination with ablation in patients with advanced hepatocellular carcinoma. J Hepatol. 2017;66(3):545‐551. doi:10.1016/j.jhep.2016.10.029 27816492 PMC5316490

[86] Xie C , Duffy AG , Mabry‐Hrones D , et al. Tremelimumab in combination with microwave ablation in patients with refractory biliary tract cancer. Hepatology. 2019;69(5):2048‐2060. doi:10.1002/hep.30482 30578687 PMC6461476

[87] Walter T , Horgan AM , McNamara M , et al. Feasibility and benefits of second‐line chemotherapy in advanced biliary tract cancer: a large retrospective study. Eur J Cancer. 2013;49(2):329‐335. doi:10.1016/j.ejca.2012.08.003 22947649

[88] Kleef R , Moss R , Szasz AM , Bohdjalian A , Bojar H , Bakacs T . Complete clinical remission of stage IV triple‐negative breast cancer lung metastasis administering low‐dose immune checkpoint blockade in combination with hyperthermia and Interleukin‐2 [published correction appears in Integr cancer Ther. 2019 Jan‐Dec;18:1534735419854816]. Integr Cancer Ther. 2018;17(4):1297‐1303. doi:10.1177/1534735418794867 30193538 PMC6247552

[89] Kleef R , Nagy R , Baierl A , et al. Low‐dose ipilimumab plus nivolumab combined with IL‐2 and hyperthermia in cancer patients with advanced disease: exploratory findings of a case series of 131 stage IV cancers ‐ a retrospective study of a single institution. Cancer Immunol Immunother. 2021;70(5):1393‐1403. doi:10.1007/s00262-020-02751-0 33151369 PMC8053148

[90] Wei Z , Yang X , Ye X , et al. Camrelizumab combined with microwave ablation improves the objective response rate in advanced non‐small cell lung cancer. J Cancer Res Ther. 2019;15(7):1629‐1634. doi:10.4103/jcrt.JCRT_990_19 31939448

[91] Matsumoto K , Yamamoto N , Hagiwara S , et al. Optimization of hyperthermia and dendritic cell immunotherapy for squamous cell carcinoma. Oncol Rep. 2011;25(6):1525‐1532. doi:10.3892/or.2011.1232 21455579

[92] Chen H , Luan X , Paholak HJ , et al. Depleting tumor‐associated Tregs via nanoparticle‐mediated hyperthermia to enhance anti‐CTLA‐4 immunotherapy. Nanomedicine (Lond). 2020;15(1):77‐92. doi:10.2217/nnm-2019-0190 31868112 PMC7132783

[93] Shi L , Wang J , Ding N , et al. Inflammation induced by incomplete radiofrequency ablation accelerates tumor progression and hinders PD‐1 immunotherapy. Nat Commun. 2019;10(1):5421. doi:10.1038/s41467-019-13204-3 31780645 PMC6883042

[94] Choi J , Lee SY . Clinical characteristics and treatment of immune‐related adverse events of immune checkpoint inhibitors. Immune Netw. 2020;20(1):e9. Published 2020 Feb 17. doi:10.4110/in.2020.20.e9 32158597 PMC7049586

[95] De Velasco G , Je Y , Bossé D , et al. Comprehensive meta‐analysis of key immune‐related adverse events from CTLA‐4 and PD‐1/PD‐L1 inhibitors in cancer patients [published correction appears in cancer Immunol res. 2018 Apr;6(4):498‐499]. Cancer Immunol Res. 2017;5(4):312‐318. doi:10.1158/2326-6066.CIR-16-0237 28246107 PMC5418853

[96] Martins F , Sofiya L , Sykiotis GP , et al. Adverse effects of immune‐checkpoint inhibitors: epidemiology, management and surveillance. Nat Rev Clin Oncol. 2019;16(9):563‐580. doi:10.1038/s41571-019-0218-0 31092901

[97] Dhawan S , Bartek J Jr , Chen CC . Cost‐effectiveness of stereotactic laser ablation (SLA) for brain tumors. Int J Hyperthermia. 2020;37(2):61‐67. doi:10.1080/02656736.2020.1774084 32672125

[98] Froelich MF , Schnitzer ML , Rathmann N , et al. Cost‐effectiveness analysis of local ablation and surgery for liver metastases of oligometastatic colorectal cancer. Cancers (Basel). 2021;13(7):1507. Published 2021 Mar 25. doi:10.3390/cancers13071507 33806059 PMC8037107

[99] Hamilton TD , MacNeill AJ , Lim H , Hunink MGM . Cost‐effectiveness analysis of cytoreductive surgery and HIPEC compared with systemic chemotherapy in isolated peritoneal carcinomatosis from metastatic colorectal cancer. Ann Surg Oncol. 2019;26(4):1110‐1117. doi:10.1245/s10434-018-07111-y 30690682

[100] de Miguel M , Calvo E . Clinical challenges of immune checkpoint inhibitors. Cancer Cell. 2020;38(3):326‐333. doi:10.1016/j.ccell.2020.07.004 32750319

[101] Kok HP , Cressman ENK , Ceelen W , et al. Heating technology for malignant tumors: a review. Int J Hyperthermia. 2020;37(1):711‐741. doi:10.1080/02656736.2020.1779357 32579419 PMC7781160

[102] Winter L , Oberacker E , Paul K , et al. Magnetic resonance thermometry: methodology, pitfalls and practical solutions. Int J Hyperthermia. 2016;32(1):63‐75. doi:10.3109/02656736.2015.1108462 26708630

[103] Notter M , Piazena H , Vaupel P . Hypofractionated re‐irradiation of large‐sized recurrent breast cancer with thermography‐controlled, contact‐free water‐filtered infra‐red‐a hyperthermia: a retrospective study of 73 patients. Int J Hyperthermia. 2017;33(2):227‐236. doi:10.1080/02656736.2016.1235731 27618745

[104] Cheng Y , Bao D , Chen X , et al. Microwave‐triggered/HSP‐targeted gold nano‐system for triple‐negative breast cancer photothermal therapy. Int J Pharm. 2021;593:120162. doi:10.1016/j.ijpharm.2020.120162 33307159

[105] Wang C , Ye Y , Hochu GM , Sadeghifar H , Gu Z . Enhanced cancer immunotherapy by microneedle patch‐assisted delivery of anti‐PD1 antibody. Nano Lett. 2016;16(4):2334‐2340. doi:10.1021/acs.nanolett.5b05030 26999507

[106] Ji Y , Winter L , Navarro L , et al. Controlled release of therapeutics from thermoresponsive nanogels: a thermal magnetic resonance feasibility study. Cancers (Basel). 2020;12(6):1380. Published 2020 May 27. doi:10.3390/cancers12061380 32471299 PMC7352924

[107] Dong X , Cheng R , Zhu S , et al. A heterojunction structured WO2.9‐WSe2 Nanoradiosensitizer increases local tumor ablation and checkpoint blockade immunotherapy upon low radiation dose. ACS Nano. 2020;14(5):5400‐5416. doi:10.1021/acsnano.9b08962 32324373

